# Gastric inhibitory polypeptide receptor: association analyses for obesity of several polymorphisms in large study groups

**DOI:** 10.1186/1471-2350-10-19

**Published:** 2009-03-02

**Authors:** Carla IG Vogel, André Scherag, Günter Brönner, Thuy T Nguyen, Hai-Jun Wang, Harald Grallert, Alexa Bornhorst, Dieter Rosskopf, Henry Völzke, Thomas Reinehr, Winfried Rief, Thomas Illig, H-Erich Wichmann, Helmut Schäfer, Johannes Hebebrand, Anke Hinney

**Affiliations:** 1 Department of Child and Adolescent Psychiatry, University of Duisburg-Essen, Essen, Germany; 2 Institute for Medical Informatics, Biometry and Epidemiology, University Duisburg-Essen, Essen, Germany; 3 Biozentrum, Universität Würzburg, Würzburg, Germany; 4 Institute of Medical Biometry and Epidemiology, Philipps-University of Marburg, Marburg, Germany; 5 Department of Maternal and Child Health, School of Public Health, Peking University, Beijing, PR China; 6 Helmholtz Zentrum München – German Research Center for Environmental Health, Institute of Epidemiology, Munich-Neuherberg, Germany; 7 Department Pharmacology, Center of Pharmacology and Experimental Therapy, Ernst-Moritz-Arndt University Greifswald, Greifswald, Germany; 8 Institute for Community Medicine, Ernst-Moritz-Arndt University Greifswald, Greifswald, Germay; 9 Vestische Hospital for Children and Adolescents, University of Witten/Herdecke, Witten, Datteln, Germany; 10 Department of Clinical Psychology and Psychotherapy, Philipps-University of Marburg, Marburg, Germany; 11 IBE, Chair of Epidemiology, University of Munich, Munich, Germany

## Abstract

**Background:**

Gastric inhibitory polypeptide (GIP) is postulated to be involved in type 2 diabetes mellitus and obesity. It exerts its function through its receptor, GIPR. We genotyped three *GIPR *SNPs (rs8111428, rs2302382 and rs1800437) in German families with at least one obese index patient, two case-control studies and two cross-sectional population-based studies.

**Methods:**

Genotyping was performed by MALDI-TOF, ARMS-PCR and RFLP. The family-study: 761 German families with at least one extremely obese child or adolescent (n = 1,041) and both parents (n = 1,522). Case-control study: (a) German obese children (n = 333) and (b) obese adults (n = 987) in comparison to 588 adult lean controls. The two cross-sectional population-based studies: KORA (n = 8,269) and SHIP (n = 4,310).

**Results:**

We detected over-transmission of the A-allele of rs2302382 in the German families (p_TDT-Test _= 0.0089). In the combined case-control sample, we estimated an odd ratio of 1.54 (95%CI 1.09;2.19, p_CA-Test _= 0.014) for homozygotes of the rs2302382 A-allele compared to individuals with no A-allele. A similar trend was found in KORA where the rs2302382 A-allele led to an increase of 0.12 BMI units (p = 0.136). In SHIP, however, the A-allele of rs2302382 was estimated to contribute an average decrease of 0.27 BMI units (p-value = 0.031).

**Conclusion:**

Our data suggest a potential relevance of *GIPR *variants for obesity. However, additional studies are warranted in light of the conflicting results obtained in one of the two population-based studies.

## Background

Obesity is a serious health problem in both developed and in developing countries. It results from an interplay of environmental and genetic factors that mediate energy intake and expenditure. From twin studies it is estimated that up to 80% of the variance in body mass index (BMI) might be genetically determined [1-4]. These genetic contributions seem to be especially important in individuals with severe or early-onset forms of obesity [5]. The molecular mechanisms of obesity are still far from being well understood.

It has been suggested that the gastric inhibitory polypeptide (GIP) may be involved in type 2 diabetes mellitus and obesity [6-8]. GIP, also known as glucose-dependent insulinotropic peptide, is a gastrointestinal hormone of 42 amino acids secreted by endocrine K cells from the duodenum in response to meal ingestion, especially for meal containing fat or glucose [7,9]. The principal action of GIP is the stimulation of glucose-dependent insulin secretion [10]. Obesity leads to insulin resistance and excessive insulin secretion after meal ingestion [11]. Plasma GIP concentrations are elevated in obese and diabetic humans and also in leptin deficient (*ob*/*ob*) rodents [11].

Variables that can lead to a dysfunction or act as antagonists of GIP have been shown to reduce obesity and insulin resistance [12]. Daily administration of GIP antagonists, such as (Pro^3^)GIP, has been able to promote weight loss and ameliorate insulin resistance in mice [11,13]. Obese patients subjected to bariatric surgery, that involves bypass of part of the small intestine, and consequently reduction of GIP secretion, presented restoration of normal glucose tolerance prior to weight loss [14].

GIP exerts its function through its specific receptor, GIPR. Inactivation of GIPR results in a defective GIP signaling [15]. Under normal diet, GIPR knockout mice (*Gipr*^-/-^) do not exhibit changes in body weight but have reduced fat mass compared with wild type (WT) mice [16,17], and normal levels of glucose and insulin [6]. Under high fat diet *Gipr*^-/- ^mice, in comparison to WT mice, have a reduced fat storage; they use fat as the main energy substrate and do not develop obesity, insulin resistance, diabetes mellitus, impaired glucose tolerance, and fatty liver like the WT [6,7,15-18]. Additionally, GIP signaling is required for effective accumulation of nutrients under high-fat diet, and inhibition of GIP signaling not only prevents obesity but also insulin resistance [19]. Recently, a study in mice [20] showed that vaccination against GIP prevents its binding to the GIP receptor, consequently reducing body weight gain under high fat diets. Nitz et al. [21] showed preliminary evidence for nominal association of a non-synonymous *GIPR *polymorphism (rs1800437) and cardiovascular disease (CVD).

Taken together, these observations show the importance of GIP signaling for fat storage rendering *GIPR *an interesting candidate for obesity. In order to investigate whether polymorphisms within the coding or adjacent regions of the *GIPR *gene are associated with obesity, we genotyped three single nucleotide polymorphisms (SNP) pertaining to the gene in up to 761 German obesity families comprising at least one extremely obese child or adolescent and both parents. These SNPs include a coding non-synonymous, conservative SNP in exon 12 (rs1800437 G/C; Glu354Gln), an intronic SNP (rs2302382 C/A in intron 1), and a SNP in the putative promoter region (rs8111428 A/G). These SNPs tag common variation of the gene (see details below). Subsequently, we attempted to validate our results in four independent German samples (two case-control studies and two cross-sectional population-based studies).

## Methods

### Characterization of Study Subjects

BMI was calculated as weight in kilograms and divided by height in squared meters. Children and adolescents with a BMI over the 90^th ^age and gender specific BMI percentile were considered overweight and children and adolescents with BMI at or over 97^th ^percentile were considered obese [22]. In adults, overweight was defined as BMI ≥ 25 kg/m^2^, obesity as BMI ≥ 30 kg/m^2 ^[23].

### Subjects

#### Obesity families

The sample consisted of German obese children, adolescents (72% had a BMI ≥ 99^th ^percentile, [22]) and their parents recruited at the Universities of Marburg and Duisburg-Essen. For the family-based association analyses we genotyped 1,041 obese children and adolescents and 1,522 of their parents (Table 1).

**Table 1:** Characteristics of participants used for family-based association, case-control and cross-sectional population-based samples in *GIPR *analyses.

	**sub-group**	**n participants**	**sex (M/F)**	**mean age (y) ± SD**	**mean BMI (kg/m)^2 ^± SD**
**Group**	**parents**	1,522	761/761	42.55 ± 5.96	30.37 ± 6.28
	children	1,041	477/564	13.88 ± 3.71	31.11 ± 6.05^1,2^
					
	obese children and				
**case-control**	adolescents	336	153/183	10.74 ± 2.75	28.29 ± 4.81^1^
	obese adults	987	361/626	46.31 ± 14.74	36.03 ± 5.39
	underweight and normal				
	weight controls	588	235/353	25.28 ± 4.41	19.34 ± 1.94
					
**KORA S3/S4**	BMI ≥ 30 kg/m^2^	1,849	876/973	54.47 ± 0.29	33.58 ± 0.08
	BMI < 30 kg/m^2^	6,420	3,267/3,153	47.92 ± 0.17	25.23 ± 0.04
					
**SHIP**	BMI ≥ 30 kg/m^2^	1,099	531/568	54.90 ± 14.34	33.55 ± 3.29
	BMI < 30 kg/m^2^	3,201	1,582/1,619	48.04 ± 16.69	25.13 ± 2.94

^1^all had a BMI ≥ the age and gender normalized 90^th ^percentile [22]

^2^72% even had a BMI ≥ the age and gender normalized 99^th ^percentile [22]

#### Case-control studies

**Cases: (A) **987 German obese (BMI ≥ 30 kg/m^2^) adults from a study at the University of Marburg sampled in the region of Marburg, Germany [24] (Table 1) and **(B) **336 overweight and obese children and adolescents (all had a BMI ≥ 90^th ^percentile) recruited within the Obeldicks program [25] at the University of Witten/Herdecke in the region of Datteln, Germany (Table 1). **Controls**: 588 normal and underweight healthy (all had BMI < 75^th ^percentile) adults who were students at the University of Marburg at the time of recruitment (Table 1, [24]). The use of lean adults who were never overweight or obese during childhood (assessed by interview [26]), as control group reduces the chances of misclassification compared to the use of lean children as controls who might become overweight in adulthood.

#### KORA

(Kooperative Gesundheitsforschung im Raum Augsburg, Surveys 3 and 4, Cooperative Health Research in the Region of Augsburg) is a cross-sectional population-based sample of 8,269 German adult individuals from the region of Augsburg (Bavaria, Germany) [27]. In KORA, 65.9% of all participants were overweight and 22.4% obese (Table 1) according to the WHO definition.

#### SHIP

(Study of Health in Pomerania) is a cross-sectional population-based survey from the Northeastern area of Germany comprising 4,310 adult German individuals. SHIP was designed to address general health and community medicine issues, with endocrine-metabolic disorders as a main focus. In SHIP, 65.8% of all participants were overweight and 25.5% obese (Table 1) according to the WHO definition.

All participants gave written informed consent and in the case of minors, their parents. The studies were approved by the local Ethical Committees and in case of KORA by the Landesärztekammer Bavaria.

### Genotyping and SNPs selection

We selected tagging SNPs (or proxy) for the two linkage disequilibrium (LD) blocks covering the coding region and the 5' region (24 Kb) of *GIPR*.

Genotyping the families for the SNPs rs2302382 and rs8111428 was performed using Matrix-assisted desorption/ionization time-of-flight mass spectrometry (MALDI-TOF MS) as described earlier [28]. SNP rs1800437 (a) was genotyped using polymerase chain reaction restriction fragment length polymorphism (PCR-RFLP) analysis. Additional genotypes for SNPs rs2302382 (b) and rs8111428 (c) were performed using ARMS-PCR as described previously (16): (a) rs1800437: F 5'-ATT ACC GGC TGA GGT GAG G-3' and R 5'-CTG GAA GGA GCT GAG GAA GA-3' digested with *Bss*SI (C-allele 245 bp, G-allele 150 and 94 bp). (b) rs2302382: F_o _5'-CAG CGT AGC TCT AGG GCA ACC GCC CGC T-3' and R_o _5'-GAT CAG GCC TGG AGG GTC CCA GGG CAA G-3':324 bp; F_i _5'-CCA CTC CGC GTG CCT CTC CCT CCT CC-3' and R_i _5'-CCG CAA CTC CCA GGC GTG ATG ATC CGT-3' (C-allele 200 bp, A-allele 177 bp). (c) rs8111428: F_o _5'-AAA GGA ACA GAC TGG AAG TAG AGA CAG-3' and R_o _5'-TTT ATG ACA CAA GCT GAA AGT CAC AC-3':486 bp; F_i _5'-TGT ATA TGA CTG TAT GTG ACT TGT GAC TG-3' and R_i _5'-CAC AAC TCT CCC TTA GTC TCA CCA AT-3' (G-allele 258 bp, A-allele 283 bp). All call rates were ≥ 99%; except for rs2302382 for the obese children and adolescents and for SHIP were the call rates were ≥ 90%.

### In silico analysis of GIPR polymorphisms

Possible alterations in splicing sites were analysed using GeneScan [29]. Potential functions were analysed using the FastSNP program [30].

### Statistical analysis

All genotype distributions were tested for deviations from Hardy Weinberg equilibrium (exact two-sided p-value >0.14). For the coding SNP rs1800437 the p-value for HWE in the parents (family study) was 0.035. Thus, we re-genotyped a 96-well plate for this SNP to exclude false genotyping results; the results were 100% identical to the initial data, thus reducing the chance of genotyping errors. Single marker family-based association analyses were carried out using the pedigree disequilibrium test (PDT-average) while FAMHAP (Version 16, [31]) and UNPHASED (Version 2.404, using the EM algorithm [32]) were used to investigate haplotypes in families. Case-control association analyses were performed using the exact or asymptotic Cochran-Armitage trend test with a linear trend or Fishers exact test for crude allele frequency comparisons. Correspondingly, BMI in KORA and SHIP were investigated by linear regression analyses assuming an additive genetic model with age and sex as covariates. Power calculations were done with the software QUANTO Version 1.2.3 [33] for common variants assuming a minor allele frequency MAF of 0.2 and α = 0.05 (two-sided). For both the family-based approach (761 trio families) and the validation sample of 1,323 cases and 588 controls the power estimates were larger than 80% to detect a log-additive genotype relative risk of 1.3. For the quantitative analyses in both population-based studies, the power estimate was larger than 80% to detect a standardized additive effect of 0.08. Thus, all samples were well powered to detect at least moderate to strong effect sizes of disease predisposing variants.

Confidence intervals were calculated with coverage of 95% (abbreviated 95%CI) and accordingly the level α for each test was 0.05 (two-sided). Unless otherwise stated all reported p-values are nominal, two-sided and not adjusted for multiple testing.

## Results

### Initial family-based association studies

**Figure 1:**
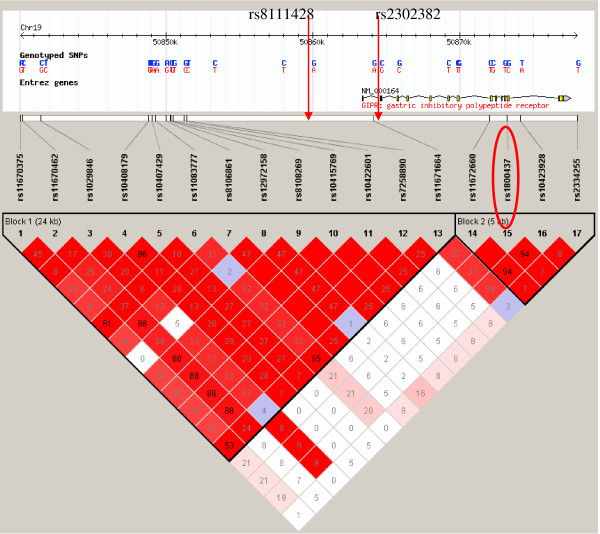
**LD structure of *GIPR***. LD structure of *GIPR *region using data from HapMap analysed by Haploview using the Spine LD algorithm. Only SNP rs1800437 is informative as LD in HapMap (ellipse). The physical localization for the other two SNPs (rs8111428 and rs2302382) was represented manually with arrows. The pairwise r^2 ^values are represented in the diamonds.

We performed family-based association analyses in up to 2,563 German Caucasian individuals from 761 families. The analyses indicated some evidence for transmission disequilibrium for the investigated markers in particular for the G-allele of rs8111428 (p-value = 0.0016) and the A-allele of rs2302382 (p-value = 0.0089), both minor alleles (Table 2). In addition, for the non-synonymous SNP rs1800437, we observed a trend for the G-allele (major allele) to be more frequently transmitted to the obese offspring (p = 0.076; Table 2). To explore if a single SNP or a haplotype was involved in obesity we further analysed haplotype structure in the gene region using the CEU population data from the International HapMap Project [34] captured by Haploview software [35] (solid spine algorithm). There are two regions of increased between-marker LD (Figure 1). The first covers 24 kb and the second 5 kb. For the two markers showing the strongest signals in our study, rs8111428 and rs2302382, there are no direct HapMap data available. However, their physical positions indicate that they could be part of the first haplotype block, as confirmed when using our family data in Haploview (data not shown). For the SNP, rs1800437, LD HapMap data was available indicating that this SNP belongs to the second region which was supported by our family data (data not shown). The pairwise r^2 ^values between the SNPs (using our family data) are shown in Table 3. Subsequently, we performed analyses of the transmitted haplotypes (Table 4). One haplotype (estimated frequency = 21%) that included the minor alleles of rs8111428 (G-allele) and rs2302382 (A-allele), was more frequently transmitted in the families (p = 0.003). Testing all haplotype combinations this haplotype had the smallest adjusted p-value of 0.0055 which is corrected for multiple testing. As the haplotype analysis revealed that no haplotype by itself leads to a stronger association signal, we decided to validate the best initial SNPs results.

**Table 2:** Results of the family-based association analyses for *GIPR *SNPs in families with severely obese offspring

**SNP**	**physical position**	**n genotyped families**	**localization**	**risk-allele**	**transmitted^1^**	**non- transmitted**	**p-value**
			Putative				
**rs8111428**	50859941	579	promoter	G	403	329	0.0016
**rs23023082**	50864409	541	Intron 1	A	395	329	0.0089
**rs1800437**	50873232	761	Exon 12	G	1,644	1,599	0.0760

^1^as derived from PDT

**Table 3:** Linkage disequilibrium (LD)^1 ^between *GIPR *SNPs of the study using the family data.

	**rs8111428^2^**	**rs2302382**
**rs2302382^2^**	0.71	-
**rs1800437^3^**	0.039	0.039

^1 ^values are given in pairwise r^2^

^2 ^No LD information in HapMap

^3 ^rs1800437 is the only proxy SNP for rs11672660 (r^2 ^= 1.0)

**Table 4:** *GIPR *haplotype analyses in families with severely obese offspring using UNPHASED (Version 2.404; using the EM algorithm) and FAMHAP (Version 16)

**rs8111428**	**rs2302382**	**rs1800437**	**transmitted**	**non- transmitted**	**estimated** **frequency of the haplotype**	**p-value**
A	A	G	18.02	30.01	0.04	0.179
A	C	G	325.20	326.90	0.57	0.919
A	C	C	103.80	129.10	0.16	0.054
**G**	**A**	**G**	**123.80**	**86.10**	**0.21**	**0.003**
G	A	C	7.20	6.90	0.01	0.179
G	C	G	8.00	7.00	0.02	0.179

The risk haplotype is marked in bold

### Validation studies in case-control samples and in cross-sectional population-based samples

To confirm the exploratory results we attempted to validate these results by case-control studies in obese adults, children, and adolescents compared to healthy normal and underweight controls. Furthermore, genotyping was done in two cross-sectional population-based samples.

The initially determined risk-allele (A) of rs2302382 was more frequent in obese children (23.8%) and adults (22.4%) than in the controls (19.1%; p_Fishers exact test _= 0.013). Using the log-additive genetic model in the joint analyses of all cases against the control group, we e.g. estimated that individuals homozygous for the A-allele have an 1.54-fold increase in the odds for obesity compared with individuals not carrying the A-allele (95%CI 1.09;2.19, p_CA-Test _= 0.014). The estimated odds ratio for the heterozygous CA carriers was 1.24 (95%CI 1.04;1.48, Table 5). When the children were analysed separately, the odds ratios were descriptively larger than those observed for the joint analyses.

Additionally, although for the non-synonymous SNP rs1800437 the major risk allele was prima vista more frequent in obese children (78.7%) and adults (79.6%) than in the controls (77.5%), there was no significant difference in allele or genotype distributions (p_Fishers exact test _= 0.395; p_CA-Test _= 0.219) (Table 5).

**Table 5:** Genotype and case-control analyses for three *GIPR *SNPs

**SNP**	**Group**	**genotypes, n (frequency)**			**frequency of allele**		**effect size estimate – odds ratio (95%CI)^1^**	**p-value^1^**
	obese children and							
**rs8111428**	adolescents	199 (0.592) AA	124 (0.369) AG	13 (0.039)GG	0.777 A	0.223 G	OR_AG _1.24 (1.04;1.49)	0.019
	obese adults	633 (0.642) AA	308 (0.312) AG	45 (0.046)GG	0.798 A	0.202 G	OR_GG _1.54 (1.07;2.33)	
	controls	396 (0.676) AA	176 (0.300) AG	14 (0.024) GG	0.826 A	0.174 G		
								
	obese children and							
**rs2302382**	adolescents	176 (0.587) CC	105 (0.350) AC	19 (0.063) AA	0.762 C	0.238 A	OR_AC _1.24 (1.04;1.48)	0.014
	obese adults	597 (0.605) CC	337 (0.341) AC	53 (0.054) AA	0.776 C	0.224 A	OR_AA _1.54 (1.09;2.19)	
	controls	385 (0.655) CC	181 (0.308) AC	22 (0.037) AA	0.809 C	0.191 A		
								
	obese children and							
**rs1800437**	adolescents	208 (0.619) GG	113 (0.336) CG	15 (0.045) CC	0.787 G	0.213 C	OR_CG _1.11 (0.94;1.32)	0.219
	obese adults	625 (0.634) GG	319 (0.324) CG	42 (0.043) CC	0.796 G	0.204 C	OR_GG _1.24 (0.88;1.73)	
	controls	355 (0.605) GG	200 (0.341) CC	32 (0.055) CC	0.775 G	0.225 C		

^1 ^exact Cochran-Armitage trend test (assuming a linear trend) not corrected for age and sex where all obese individuals were pooled; separate comparison of adults and children against the controls revealed no substantially different results

The cross-sectional population-based samples KORA and SHIP were both genotyped for SNP rs2302382. In KORA, the regression analyses showed a trend for the presence of one A-allele to lead to an average increase of 0.12 BMI units (95%CI -0.04;0.28; p-value = 0.136) (Table 6). Similarly, stratification by WHO BMI categories underlined this trend (p_CA-Test; asy _= 0.037 for the comparison of BMI < 30 vs. BMI ≥ 30). In SHIP, however, the effect of the A-allele pointed into the opposite direction. In contrast to all previous findings, the A-allele of rs2302382 was estimated to decrease mean BMI units by 0.27 kg/m^2 ^(95%CI -0.52; -0.24; p-value = 0.031; p_CA-Test;asy _= 0.039 for the comparison of BMI <30 vs. BMI ≥ 30 kg/m^2^).

**Table 6:** Genotype results for *GIPR *SNP rs2302382 in the population-based cohorts KORA and SHIP

**Group**		**genotypes, n (frequency)**			**Allele frequency**		**effect size estimate (95%CI)^1^**	**p-value^1^**
		**CC**	**CA**	**AA**	**C**	**A**		
**KORA**	BMI ≥ 30 kg/m^2^	1,103 (0.597)	644 (0.348)	102 (0.055)	0.771	0.229	β_A _0.12	0.136
	BMI <30 kg/m^2^	3,993 (0.622)	2,117 (0.330)	310 (0.048)	0.787	0.213	(-0.04; 0.28)	
								
**SHIP**	BMI ≥ 30 kg/m^2^	645 (0.640)	332 (0.329)	31 (0.031)	0.805	0.195	β_A _-0.27 (-0.52; -0.24)	0.031
	BMI <30 kg/m^2^	1,842 (0.622)	995 (0.336)	126 (0.042)	0.790	0.210		

^1 ^linear regression under an additive genetic model (risk-allele A) corrected for age and sex, two-sided p-value

### Predictions of putative functional consequences of GIPR polymorphisms

The intronic SNP rs2302382 was analysed for alternative splice sites (using GeneScan) caused by the base change, but no respective alterations were found. No known function was found in FastSNP for rs2302382.

The non-synonymous SNP Glu354Gln (rs1800437) is located in a transmembrane domain of GIPR [21]. Analysis of the SNP rs1800437 using FastSNP showed that the predicted altered protein has a low to medium risk to be *damaging *(ranking 2–3; whereby 5 is the maximum risk). This tool predicts that the respective mutation G/C creates an additional exonic splicing enhancer in the sequence comprising the C-allele.

## Discussion

We provide evidence for an association of polymorphisms in and near the *GIPR *gene with obesity or increased BMI. We performed family-based association tests in up to 761 German nuclear families with severely obese offspring. The haplotype analyses in the genotyped region indicated the presence of two distinct regions of increased linkage disequilibrium (LD): first, a putative regulatory region for *GIPR *comprising SNPs rs8111428 and rs2302382 and second, a region covering the *GIPR *coding region comprised SNP rs1800437.

Evidence for association with obesity was found for the SNPs in the putative regulatory region of *GIPR*. In nuclear families, we observed an increased transmission rate for the minor alleles of rs8111428 (G-allele) and rs2302382 (A-allele) to the obese offspring, indicating the presence of a possible risk haplotype for obesity. Further analyses in two independent samples of cases and one control sample underlined and validated this association. In two large population-based cross-sectional samples of adults, however, the results were ambiguous. While we observed a similar trend for rs2302382 in KORA (> 8,000 individuals) where the risk allele was related to an increased BMI, the same risk allele was inversely associated with BMI in SHIP.

Analyses of the putative regulatory region of *GIPR *were previously performed in patients with Cushing syndrome, which is associated with obesity [36,37]. There were no significant differences in genotype frequencies between patients and controls [36]. Our family and case-control data suggested an increased obesity risk with an estimated odds ratio of 1.54 for individuals with two copies of the risk A-allele at SNP rs2302382 in the putative regulatory region. Since this SNP was not directly analysed in previous reports [36,37], functional studies are warranted. All participants were recruited in Germany, for which population stratification effects have shown to be of minor importance under a case-control design [38].

Additionally, in the second LD region we found a trend for the G-allele of rs1800437 to be more frequently transmitted to obese children. The same allele was also more frequently detected in obese cases than in controls (Tables 2 and 5).

Despite all evidence for the involvement and importance of *GIPR *in obesity, until now few studies analysed variants in *GIPR *and their risk for obesity. Some studies involving the non-synonymous variant rs1800437 did not reveal association with increased BMI [21,39] or to non-insulin-dependent diabetes [39,40]. However, in one study, C-peptide concentrations in serum of homozygous individuals for the C-allele (minor allele) of rs1800437 were significantly decreased (14%) after fasting [39]. Additionally, association with lower cholesterol levels was found in heterozygous individuals with CVD [21]. Thus, the results of both studies (Lower C-peptide concentrations [39] and lower cholesterol levels [21]) are in line with our case-control study where the C-allele was more frequent in controls than in cases. Additionally, in our German obesity families, we found a trend of the G-allele (major allele) to be more frequently transmitted to severely obese offspring (p = 0.076). Taken together, these results suggest an association of the G-allele of rs1800437 with obesity. If a dysfunctional GIPR receptor leads to a lower fat mass we have to assume that obesity would be associated with gain of function mutations. Hence, we assume a gain of function for the G-allele of non-synonymous SNP rs1800437 and the respective risk alleles of the SNPs in the putatively regulatory region (rs2302382 and rs8111428) which might alter transcription binding sites causing an increased gene expression. Investigations with independent and large samples are necessary to validate our observed associations. Samples from the extremes of the phenotype would be the best choice. Once an even more robust signal is obtained, re-sequencing as well as functional studies will be necessary to elucidate the functional role of the *GIPR *variants.

*GIPR *is located at the region on chromosome 19q13 that was reported to have highly differentiated SNPs showing strong geographical variation within the English population [41]. KORA and SHIP are population-based samples with individuals from Southern and Northern Germany. A similar effect as in the English population it could, at least in theory, account for the discrepant result in SHIP. An exploration of differences in genotype frequencies of rs2302382 in KORA and SHIP, irrespective of phenotype, indicated that this might be the case (p = 0.035). Additionally, there are multiple phenotypic variables which differ between KORA and SHIP; examples are hypertension [42] or smoking behaviour [43], which could also account for the different genotypic effect.

It might seem surprising that *GIPR *has not been detected in any of the currently published genome-wide association studies on obesity (BMI) [e.g. [26,44-48]]. There are at least two explanations: First, genome-wide SNP chips do not cover well the region of *GIPR*. For example, the Affymetrix Genome-Wide Human SNP 6.0 Array (with more than 906,600 SNPs) in the region ± 50 Kb of *GIPR *only comprises 3 SNPs and none of these SNPs is within *GIPR*. The Illumina 580 K array on the other hand comprises 19 SNPs in the region ± 50 Kb of *GIPR*, but only three of them are within the gene. One idea would be to use imputation analyses to solve this problem. Imputations, however, heavily rely on just a few HapMap individuals and the assumption that linkage disequilibrium between markers is the same for these individuals and the individuals actually genotyped. Moreover, for markers like rs8111428 and rs2302382 there is no HapMap information available making imputations impossible. Second, current meta-analyses of genome-wide association studies did focus on BMI in the general population; it might well be that GIPR variants have a major impact in extremes of the phenotype only.

## Conclusion

In conclusion, our data provide a first step towards identification of *GIPR *variants potentially involved in obesity. Most likely variations in the putative regulatory region of the gene (e.g. rs2302382) are the most promising candidates for independent validations in case-control samples or in selected family samples as well as ultimately in functional studies. If our findings indeed are truly positive, this study also supports the observation [26,49] that cross-sectional population-based studies seem to be less powerful to detect obesity-marker associations as they are rarely enriched with extremely obese individuals.

## Competing interests

The authors declare that they have no competing interests.

## Authors' contributions

CIGV participated of the study design, perform the genotyping and drafted the manuscript; AS participated of the study design, performed statistical analysis and drafted the manuscript. TTN performed statistical analysis. AB and HG performed genotyping. GB, HJW, DR, HV, TR, WR, TI, and HEW participated of the study design; JH and AH conceived the study, participated in its design and coordination and drafted the manuscript. All authors read and approved the final manuscript.

## Pre-publication history

The pre-publication history for this paper can be accessed here:


